# Lower-limb muscle strength: normative data from an observational population-based study

**DOI:** 10.1186/s12891-020-3098-7

**Published:** 2020-02-08

**Authors:** Julie A. Pasco, Amanda L. Stuart, Kara L. Holloway-Kew, Monica C. Tembo, Sophia X. Sui, Kara B. Anderson, Natalie K. Hyde, Lana J. Williams, Mark A. Kotowicz

**Affiliations:** 1 School of Medicine, Deakin University, Geelong, VIC 3220 Australia; 2 Department of Medicine – Western Health, The University of Melbourne, St Albans, Australia; 3 Department of Epidemiology and Preventive Medicine, Monash University, Melbourne, Australia; 4 Barwon Health, Geelong, Australia

**Keywords:** Specific force, Manual muscle test, Dynapenia, Reference values, Sarcopenia, Hand-held dynamometry

## Abstract

**Background:**

The extent of muscle deterioration associated with ageing or disease can be quantified by comparison with appropriate reference data. The objective of this study is to present normative data for lower-limb muscle strength and quality for 573 males and 923 females aged 20-97 yr participating in the Geelong Osteoporosis Study in southeastern Australia.

**Methods:**

In this cross-sectional study, measures of muscle strength for hip flexors and hip abductors were obtained using a Nicholas manual muscle tester, a hand-held dynamometer (HHD; kg). Leg lean mass was measured by dual energy x-ray absorptiometry (DXA; kg), and muscle quality calculated as strength/mass (N/kg).

**Results:**

For both sexes, muscle strength and quality decreased with advancing age. Age explained 12.9–25.3% of the variance in muscle strength in males, and 20.8–24.6% in females; age explained less of the variance in muscle quality. Means and standard deviations for muscle strength and quality for each muscle group are reported by age-decade for each sex, and cutpoints equivalent to T-scores of − 2.0 and − 1.0 were derived using data from young males (*n* = 89) and females (*n* = 148) aged 20–39 years.

**Conclusions:**

These data will be useful for quantifying the extent of dynapenia and poor muscle quality among adults in the general population in the face of frailty, sarcopenia and other age-related muscle dysfunction.

## Background

Age-related loss of skeletal muscle strength, or dynapenia [1], is a hallmark of impairment that affects the health and wellbeing of older individuals. Muscle strength is important for mobility [2] and other activities of daily living [3], and is central for maintaining independence in older age. Muscle weakness is a predictor for falls [4], falls-related hospitalisation [5], fractures [6], comorbidities such as the metabolic syndrome [7] and all-cause mortality [8]. Weakness is one of five physical characteristics considered by Fried et al. [9] to support a diagnosis of frailty, and low muscle strength is a key component of sarcopenia [10–13].

The extent of muscle deterioration associated with ageing, injury or disease can be gauged with reference to appropriate normative data. We have previously reported normative data for total and appendicular lean mass with and without adjustment for height [14] and body mass index (BMI) [15]. These surrogate measures of muscle mass have been incorporated into different definitions for sarcopenia from Europe [10, 11] and the USA [12] and yet are applied to the Australian population where local cutpoints might have relevance.

Measures of handgrip strength are often recommended in the assessment of both sarcopenia and frailty, and reference ranges have been published for populations in Australia [16] and elsewhere [17]. However, lower-limb rather than upper-limb weakness specifically compromises functional capacities [18] and increases falls risk [19]. Although there is evidence that handgrip strength is indicative of overall muscle strength [20], loss of maximal strength is not consistent across all muscle groups [21] and good agreement between handgrip and lower limb strength is not supported in all studies [22]. Furthermore, assessment of lower limb strength offers an alternative when handgrip strength is not feasible due to hand disability. A role for measuring lower-limb muscle strength in geriatric assessment needs the support of appropriate normative data for quantifying deficits, but there are few published for the lower-limb [23].

Muscle strength deteriorates more rapidly and to a greater extent than muscle mass during ageing, and this divergence is suggestive of an ageing-related loss of muscle quality [24, 25]. Muscle quality is generally conceptualised as muscle strength or power per unit of muscle mass [18] and, in this study, we refer to muscle quality as the ratio of muscle strength per unit of lean mass. The aim of this cross-sectional, population-based study of adults was to provide age- and sex-specific norms for skeletal muscle strength and quality in the lower limbs, specifically for the muscle groups known as hip flexors and hip abductors.

## Methods

### Participants

Data for this cross-sectional sub-study were generated by the Geelong Osteoporosis Study (GOS), a population-based cohort study in the Barwon Statistical Division in southeastern Australia. Details of study design, participation and retention are described elsewhere [26]. Age-stratified samples of males and females were drawn at random using the electoral roll as the sampling frame. In Australia, registration with the Australian Electoral Commission is compulsory for adults aged 18 years and over, so the electoral roll provides a comprehensive listing of all residents. A listing on the electoral roll encompassing the Barwon Statistical Division fulfilled eligibility criteria. Participants were excluded if they had resided in the region for less than 6 months or were unable to provide written, informed consent. In total, 1467 males were recruited 2001–2006 (ages 20–96 years, 67% participation) and 1494 females were recruited 1993–1997 (ages 20–93 years, 77% participation). This study utilises data for 573 males from their baseline assessment and 882 females from their 6-year follow-up assessment. The cohort composition was 98.2% Caucasian, 0.8% Asian, 0.2% Indian, 0.1% Indigenous and 0.8% other or unknown ethnicity.

### Muscle strength assessment

A break test technique was utilised to measure peak eccentric muscle strength in the legs using a hand-held dynamometer (HHD), the Nicholas manual muscle tester (model 01160, Lafayette Instrument Company). The HHD records the peak force required to break an isometric contraction. This technique has been identified previously as a reliable method for assessing lower-limb muscle strength in adults [27, 28]. The procedure was explained to participants before the tests commenced; no warm-ups or practice attempts were trialed. The examiner did not stabilise the participant during testing but provided verbal encouragement. For the measurement of hip flexion strength, the participant was seated with feet hanging above floor level. With the test thigh held 10 cm above the table surface, the HHD was positioned 5 cm proximal to the patella and the contralateral limb was neutral. The examiner applied a downward force on the test thigh while the participant resisted, until resistance could no longer be maintained. For the measurement of hip abduction strength, the participant was in a side-lying position, with the test leg outstretched and raised 20 cm above the surface of the bench; the HHD was positioned 10 cm proximal to the lateral malleolus. Measurements were repeated bilaterally, in triplicate for hip flexors (for 573 males and 922 females) and hip abductors (for 565 males 916 females). There was no recovery period between trials. Multiplying the maximal registered value (kg) by 9.81 converted the strength to Newtons (N). Values for missing data were not imputed. The HHD was calibrated by the manufacturer before each follow-up phase.

### Muscle mass assessment

Lean soft tissue mass of the legs was measured for 568 males and 914 females using whole body dual energy x-ray absorptiometry (DXA; DPX-L, Lunar, Madison, WI, USA) as previously described [14]. We used the standard segmentation of whole body DXA scans into axial (head, spine, trunk and pelvis) and appendicular (arms and legs) regions using the predefined whole body model as required by the software. The legs were isolated using cut-lines that passed through the femoral necks. DXA-derived lean soft tissue mass comprises non-fat and non-bone tissue that correlates well with muscle mass measured using magnetic resonance imaging (MRI) in males and females [29, 30].

### Muscle quality assessment

In this study, muscle quality was calculated as the ratio of lower limb muscle strength to DXA-derived leg lean mass (N/kg). This approach is similar to that employed in other studies [31, 32]. Muscle quality was determined for each muscle group (hip flexors and hip abductors) separately for each leg and the maximum for each muscle group was used in analyses.

### Other measures

Body mass was measured to ±0.1 kg using electronic scales, standing height was measured to ±0.01 m using a wall-mounted stadiometer and BMI calculated as body mass/height^2^ (kg/m^2^). Participants were not fasted prior to being measured. All clinical measures were performed by trained personnel.

### Statistical analysis

Data for males and females were analysed separately. For each muscle group on each side, muscle quality was calculated as muscle strength referenced to leg lean mass (N/kg). Sex-specific means and standard deviations (SD) for muscle strength and quality were calculated for all participants (and expressed for age-decades 20–29 to 70–79 years, and 80+ years) and for a young adult reference sample aged 20–39 years, which corresponds to the reference sample used for lean mass [14]. Cutpoints were derived using young adult reference data and were equivalent to T-scores of − 2.0 and − 1.0.

Linear regression models were developed to examine the associations between muscle strength (and muscle quality) of each muscle group and age, body mass and height. Age was centered around the mean. The selection for parsimonious models for muscle strength and muscle quality involved maximising the coefficient of determination (R^2^) while minimising the Mallow’s Cp statistic. Statistical analyses were performed using Minitab (version 16, Minitab, State College, PA, USA).

## Results

Characteristics of all participants are shown in Table 1.

**Table 1:** Participant characteristics. Data are displayed as median (interquartile range) or mean ± standard deviation

	Males (*n* = 573)	Females (*n* = 923)
Age (yr)	55.6 (45.6–66.7)	58.1 (44.3–71.6)
Body mass (kg)	83.7 ± 13.7	70.7 ± 15.2
Height (m)	1.74 ± 0.07	1.60 ± 0.07
Body mass index (kg/m^2^)	27.5 ± 4.1	27.5 ± 5.7
Appendicular lean mass (kg)	26.3 ± 3.6	17.2 ± 2.5
Relative appendicular lean mass (kg/m^2^)	8.63 ± 0.90	6.68 ± 0.81

Young adult reference data were derived from 89 males and 148 females for maximum muscle strength, and 89 males and 145 females for maximum muscle quality, for ages 20–39 years. These data are shown in Table 2 together with cutpoints corresponding to T-scores of − 1 and − 2 for hip flexors and hip abductors.

**Table 2:** Young adult (20–39 years) reference data for hip flexors and hip abductors strength (N) and muscle quality (N/kg) together with cutpoints equivalent to T scores of − 1.0 and − 2.0

	Males (*n* = 89)		Females (*n* = 148)	
	Hip Flexors	Hip Abductors	Hip Flexors	Hip Abductors
Muscle strength				
Mean ± SD	342 ± 73	203 ± 49	200 ± 51	151 ± 56
T score = − 1.0	269	154	149	98
T score = − 2.0	196	105	96	40
Muscle quality^a^				
Mean ± SD	35.7 ± 6.7	21.2 ± 4.7	30.9 ± 9.0	23.4 ± 8.5
T score = − 1.0	29.0	16.5	21.9	14.9
T score = − 2.0	22.4	11.8	13.0	6.4

^a^Missing data: *n* = 3 females

Sex-specific means and SDs for maximum muscle strength and muscle quality values for each age decade are shown in Table 3. For each group of muscles, an age-related decline was evident across the age range. The age-related decline observed in muscle quality was less marked and less consistent than for muscle strength.

**Table 3:** Leg muscle strength (N) and muscle quality (N/kg) for men and women by 10-year age group and for the full age range (20–98 years). Data are displayed as mean ± standard deviation

Age group (yr)	Males			Females		
	n	Hip Flexors^a^	Hip Abductors^b^	n	Hip Flexors^a^	Hip Abductors^b^
Muscle strength						
20–29	17	324 ± 61	188 ± 33	23	199 ± 57	142 ± 40
30–39	72	346 ± 76	207 ± 52	125	201 ± 50	153 ± 58
40–49	118	323 ± 64	195 ± 50	174	182 ± 49	161 ± 53
50–59	139	306 ± 66	192 ± 56	185	165 ± 48	146 ± 51
60–69	112	263 ± 72	180 ± 54	160	157 ± 46	129 ± 39
70–79	93	232 ± 65	148 ± 45	164	133 ± 45	108 ± 41
80+	22	229 ± 71	144 ± 43	91	112 ± 38	84 ± 31
All	573	292 ± 78	183 ± 54	922	161 ± 54	134 ± 53
Muscle quality						
20–29	17	35.4 ± 5.5	20.3 ± 3.0	23	31.8 ± 14.7	22.4 ± 9.2
30–39	72	35.8 ± 6.9	21.4 ± 5.0	122	30.8 ± 7.5	23.6 ± 8.4
40–49	118	34.4 ± 7.4	20.6 ± 5.2	172	28.4 ± 7.4	25.1 ± 7.9
50–59	139	33.1 ± 6.9	20.8 ± 5.9	185	26.2 ± 7.2	23.2 ± 7.8
60–69	112	29.8 ± 7.6	20.4 ± 5.6	159	26.1 ± 8.1	21.4 ± 6.8
70–79	93	27.7 ± 7.6	17.6 ± 5.2	162	22.6 ± 7.4	18.4 ± 7.0
80+	22	27.4 ± 7.8	17.7 ± 5.2	90	21.0 ± 7.4	15.7 ± 5.4
All	573	32.0 ± 7.8	20.1 ± 5.5	913	26.2 ± 8.3	21.9 ± 7.9

Missing data for muscle strength: ^a^ *n* = 1 female; ^b^ *n* = 8 males and *n* = 7 females

Missing data for muscle quality: ^a^ *n* = 5 males and *n* = 10 females; ^b^ *n* = 13 males and *n* = 16 females

**Fig. 1:**
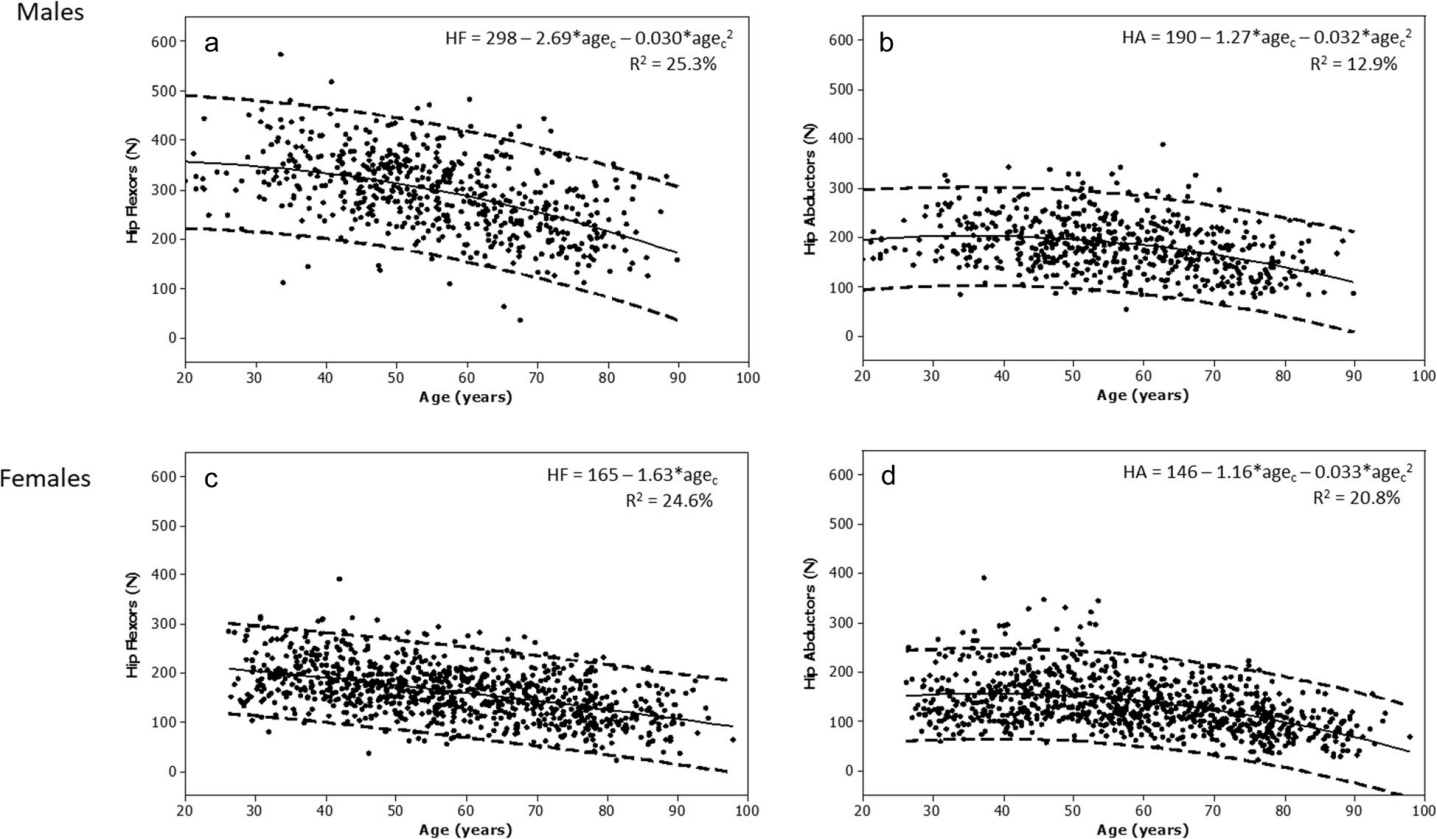
The association between age and muscle strength of the hip flexors for males and females (**a** and **c**), and  hip abductors for males and females (**b** and **d**). Regression line (solid) and 95% prediction interval (dashed), regression equations and adjusted R^2^ values are shown. Abbreviations: HF hip flexors; HA hip abductors; Age_c_ centred (mean 55.7 yr)

The relationship between maximum muscle strength and age was curvilinear for males; for females, a curvilinear pattern was also observed for the hip abductors, but for hip flexors, the relationship was linear (Fig. 1). Age explained 12.9–25.3% of the variance in muscle strength in males, and 20.8–24.6% in females. There was a weak positive correlation between muscle strength and body mass; for males the correlations were 0.19 for hip flexors and 0.22 for hip abductors, and for females, 0.21 for hip flexors and 0.24 for hip abductors (all *p* < 0.001). There was a weak positive correlation between muscle strength and height; for males *r* = 0.28 for hip flexors, and *r* = 0.20 for hip abductors, and for females, *r* = 0.27 for hip flexors and 0.28 for hip abductors (all *p* < 0.001). Correlations between muscle strength and BMI were also weak and positive; for males *r* = 0.16 for hip flexors (*p* = 0.058) and *r* = 0.13 for hip abductors (*p* = 0.002), and for females, *r* = 0.11 for hip flexors (*p* = 0.001) and 0.14 for hip abductors (*p* < 0.001). Best models for predicting muscle strength are shown in Table 4.

**Table 4:** Constant values, regression coefficients and adjusted coefficients of determination (R^2^) for linear regression models for muscle strength (N) and muscle quality (N/kg) for hip flexors and hip abductors

	Constant	Age-c^a^	(Age-c^a^)^2^	Body mass	Height (m)	Adjusted R^2^ (%)
Muscle strength						
Males						
Hip flexors	3.4	−2.52	−0.0241	0.666	136	28.9
Hip abductors	125	−1.25	−0.0252	0.754		16.3
Females						
Hip flexors	129	−1.56		0.512		26.6
Hip abductors	24.6	−1.05	−0.0266	0.482	53.2	23.2
Muscle quality						
Males						
Hip flexors	59.3	−0.211		−0.0598	−12.8	16.2
Hip abductors	44.2	−0.0903	−0.00272		−13.5	7.4
Females						
Hip flexors	66.1	−0.227			−24.6	16.4
Hip abductors	44.2	−0.150	−0.00502		−13.0	14.3

Data represent associations with age (yr) centred about the mean (55.7 yr), body mass (kg) and height (m)

All regression coefficients are significant at *p* < 0.001

When muscle strength was expressed as a ratio to body mass, the correlation with age for males was *r* = − 0.48 for hip flexors and *r* = − 0.32 for hip abductors (*p* < 0.001); the correlations were strengthened when muscle strength was scaled to body mass raised to two-thirds, *r* = − 0.50 for hip flexors and *r* = − 0.34 for hip abductors (*p* < 0.001). Similarly, for females, when muscle strength was expressed as a ratio to body mass, the correlation with age was *r* = − 0.42 for hip flexors and *r* = − 0.37 for hip abductors (*p* < 0.001); and when muscle strength was scaled to body mass raised to two-thirds, *r* = − 0.46 for hip flexors and *r* = − 0.10 for hip abductors (*p* < 0.001).

**Fig. 2:**
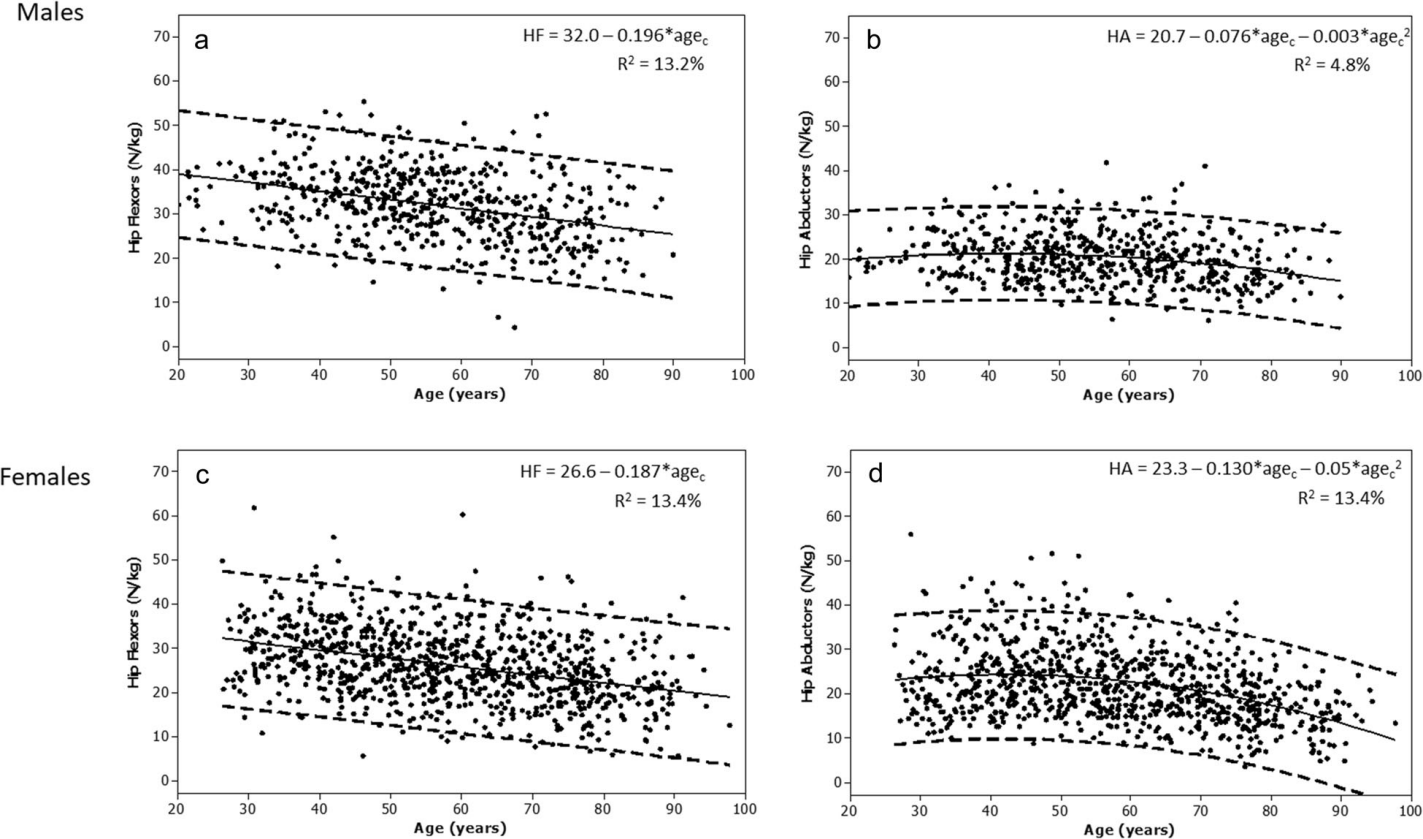
The association between age and muscle quality of the hip flexors for men and women (**a** and **c**), and hip abductors for men and women (**b** and **d**). Regression line (solid) and 95% prediction interval (dashed), regression equations and adjusted R^2^ values are shown. Abbreviations: HF hip flexors; HA hip abductors; Age_c_ centred (mean 55.7 yr)

For muscle quality, the relationship with age explained less of the variance in muscle quality (Fig. 2). For males, muscle quality was weakly and negatively associated with body mass for the hip flexors and abductors (*r* = − 0.15, *p* < 0.001; *r* = − 0.09, *p* = 0.04, respectively). For females, the correlation between muscle quality and body mass was poor (hip flexors *r* = − 0.002, *p* = 0.9 and hip abductors *r* = 0.07, *p* = 0.05). Correlations between muscle quality and height were inconsistent: for males, hip flexors (*r* = − 0.06, *p* = 0.13) and hip abductors (*r* = − 0.12, *p* = 0.007), and for females, hip flexors (*r* = − 0.01, *p* = 0.8) and hip abductors (*r* = 0.07, *p* = 0.05). For males, negative weak correlations were also observed between muscle quality and BMI for hip flexors (*r* = − 0.13, *p* = 0.002) but not for hip abductors (*r* = − 0.04, *p* = 0.350). For females, no correlations were detected between hip flexor quality and BMI (*r* = − 0.00, *p* = 0.990) or hip abductor quality and BMI (*r* = 0.04, *p* = 0.221). Best models for predicting muscle quality are shown in Table 4.

## Discussion

Here we provide sex-specific normative data describing muscle strength and muscle quality for hip flexors and hip abductors for males and females from Australia. The overall and age-specific data are useful for calculating T-scores and Z-scores and for quantifying the extent of dynapenia among adults in the general population. These data can be used in conjunction with measures of muscle mass to determine muscle quality and with performance for identifying conditions such as sarcopenia and frailty.

We report generally weak correlations between muscle strength and body mass or height. Despite being statistically significant, the low correlation coefficients suggest little relationship between these variables. However, it is recognised that body size influences muscle strength [12, 33]. Instead of a linear adjustment for body mass, allometric scaling of muscle strength to body mass raised to the power of two-thirds has been recommended as an effective approach to account for the effects of body size on muscle strength [33]. Our results support this concept, as the linear correlations between muscle strength and age were strengthened when muscle strength was normalised for body mass raised to the power of two-thirds.

Loss of lower-limb strength causes problems with locomotion and activities of daily living [3]. Hip abductor and hip extensor muscles can work together to affect postural reactions and are important for standing and walking [34]. A recent systematic review [23] highlighted a study by Andews at al [35] that utilised HHD to test lower-limb strength for males and females aged 50–79 years. Participant positioning for hip abduction strength testing was similar to the method we employed; however, matching for sex-and age-decade, our muscle strength values were approximately one-third lower than their maximum values for dominant or non-dominant sides. Further, our correlations between hip abduction strength and body mass or height were lower than their reported pooled values for males and females. In contrast to our study, their convenience sample of 156 adults was smaller and recruited from a population in the USA, a series of health-related exclusions retained healthy participants only and maximal muscle strength was tested using a different type of dynamometer. Such disparities in study design could have contributed to the reported differences in muscle strength and correlations with body mass and height.

Baseline data from a randomised controlled trial, conducted in a similar region of Australia to this study, involving 90 females aged ≥70 years, used a comparable method for measuring lower-limb strength (except the mean of three trials on the left side was routinely calculated) to report overall median values of 11.7 kg for hip flexion strength and 8.0 kg for hip abduction strength [36]. These values for selected trial participants were similar, albeit lower, than the median (95% confidence interval) maximal values of 12.4 (11.9, 12.9) kg and 9.8 (9.3, 10.3) kg, respectively, for 253 females aged 70 years and older reported in this study. Using mean values for one side instead of the maximum of both sides, could account for the minor differences noted between these studies.

In contrast to this study involving population-based data, group-specific normative data may be more relevant for individuals with different musculature, such as elite athletes. Based on data from a cohort study of 350 healthy, elite female handball and football players in Norway, normative data were established for several isokinetic concentric knee extension and flexion muscle strength tests performed bilaterally using a dynamometer [37]. Differences in muscle strength were detected between the handball and football players. For these athletes, it was important to identify differences between right and left sides and also between agonist-antagonist muscle groups, as strength asymmetries have been implicated in injuries [38, 39]. It is clear that for these females, normative data developed from an appropriate population are important as these data could be useful for setting goals for muscle strength rehabilitation following injury.

The strength of our study is that participants were selected using a random process from the electoral rolls, rather than from convenience samples or on the basis of disease. We utilised objective measures of muscle strength, lean mass and anthropometry; however, inter-individual variability of testing could have caused some discrepancies with the data collected. It is possible that maximum performance on the muscle strength tests could have been influenced by sub-optimal performance by some participants and, as there was no recovery between trials, fatigue in later trials may have limited maximal readings. We recognise that the use of lean mass may be imprecise as a surrogate measure of muscle mass, as differences in muscle composition relating to factors such as fat infiltration, lean tissue thickness and hydration may not have been captured by DXA. Further, DXA scans in the non-fasted state could over-estimate lean mass. These limitations may have influenced the validity of muscle quality estimates. Normative data have been presented by age for males and females and we have not accounted for differences in body size. In this study, the term ‘muscle quality’ was estimated using muscle strength of particular muscle groups in relation to leg lean mass of the whole leg rather than individual muscle groups. Data presented here are representative of the underlying population, as participants were not excluded because of exposure to medications or disease. As the sample is from Australia, and comprises mainly Caucasians, the data might not be generalisable to other populations or other ethnicities.

## Conclusion

The data presented here will be useful for quantifying the extent of dynapenia and poor muscle quality for males and females in the general population in the face of frailty, sarcopenia and other age-related skeletal muscle function deficits.

## Data Availability

The datasets used and/or analysed during the current study are available from the corresponding author on reasonable request.

## Abbreviations

BMI: Body mass index
DXA: Dual energy x-ray absorptiometry
GOS: Geelong Osteoporosis Study
HA: Hip abductors
HF: Hip flexors
HHD: Hand-held dynamometer
MQ: Muscle quality
SD: Standard deviation
